# 遗传性铁粒幼细胞贫血新突变一例报告及文献复习

**DOI:** 10.3760/cma.j.issn.0253-2727.2021.07.013

**Published:** 2021-07

**Authors:** 潇 韩, 钦 文, 学 刘, 锴 万, 红菊 颜, 诚 张, 曦 张

**Affiliations:** 陆军军医大学新桥医院血液病医学中心，创伤、烧伤与复合伤国家重点实验室，全军血液病中心，重庆市医学重点学科，重庆 400037 Medical Center of Hematology, Xinqiao Hospital of Army Medical University, State Key Laboratory of Trauma, Burns and Combined Injury, PLA Blood Disease Center, Chongqing Key Discipline of Medicine, Chongqing 400037, China

X连锁铁粒幼细胞贫血（XLSA）是遗传性铁粒幼细胞贫血（CSA）最常见类型（约占40％）[1]，由位于Xp11.21染色体上的红系特异性5′-氨基乙酰丙酸合酶2（ALAS2）基因突变引起并表现出X连锁遗传模式[2]。XLSA特征为骨髓中无效红细胞生成、大量的环形铁粒幼红细胞、小细胞低色素性贫血以及血清铁和铁蛋白水平升高[3]。目前，已鉴定出104种不同的ALAS2突变。本研究中，我们报道了一个新发现的ALAS2基因V110Afs*2移码突变并进行文献复习如下。

## 病例资料

患者，男，16岁，因“反复头昏、乏力2年”于2019年10月15日就诊我院。患者2年前开始反复出现头晕、乏力，当地医院查血常规提示小细胞低色素性贫血并予对症治疗，症状无改善，需反复输血，为求进一步诊治来我院。查体：重度贫血貌，全身皮肤黏膜无黄染及出血，浅表淋巴结未触及肿大，胸骨无压痛，心肺未见明显异常，肝脾肋缘下未触及，双下肢无水肿。既往史及家族史无特殊。血常规：WBC 5.58×10^9^/L、RBC 2.5×10^12^/L、HGB 51 g/L、平均红细胞体积（MCV）70 fl、平均血红蛋白含量（MCH）20.4 pg、平均血红蛋白浓度（MCHC）291 g/L、红细胞分布宽度（RDW）38.8％、网织红细胞绝对计数（Ret）3.3×10^9^/L、PLT 858×10^9^/L；铁蛋白：>2000 µg/L；血清铁：34.1 mmol/L；外周血涂片：无核红细胞形态大小不一，中心淡染区扩大，椭圆、泪滴、棒状、碎片等异形红细胞可见（图1A）；骨髓象：增生活跃，粒系占0.280，红系占0.675，粒红比值为0.41，红系增生活跃，以中晚幼红细胞增生为主，形态可见体积小、胞质蓝染、核固缩，淋巴细胞占4.5％，全片见巨核细胞48个，环状铁粒幼红细胞增多（69％），细胞内铁81％，细胞外铁（++），红细胞形态提示小细胞低色素，考虑环形铁粒幼细胞贫血（图1B）。骨髓活检：增生性骨髓象（红系比例增高）。流式免疫分型：粒系减低，红系比例增高，可见CD71减弱。FISH：5号、7号、8号、20号、性染色体未见异常。染色体核型：46,XY[10]。

**图1:**
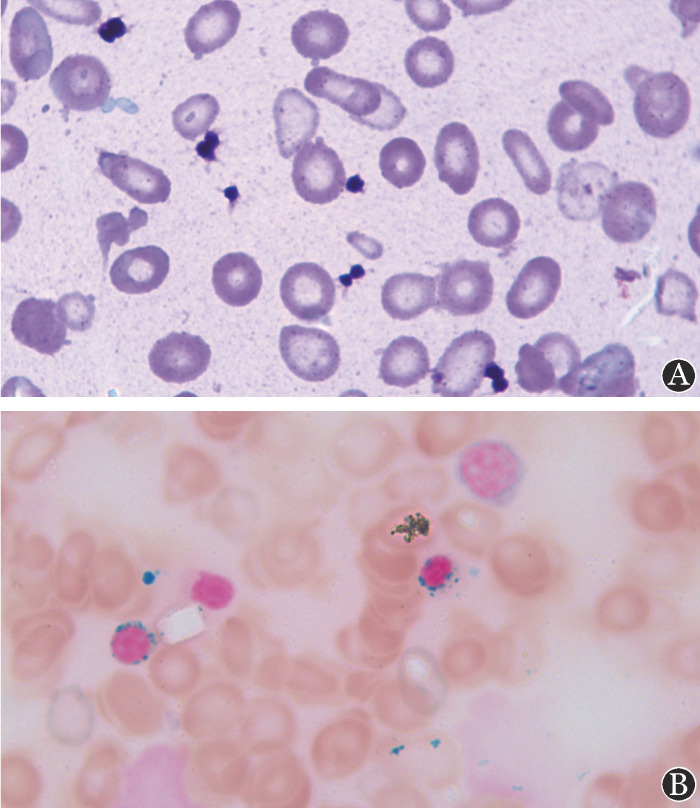
遗传性铁粒幼细胞贫血患者外周血涂片及骨髓铁染色结果 A：外周血涂片示无核红细胞形态大小不一，中心淡染区扩大，可见椭圆、泪滴、棒状、碎片等异形红细胞；B：骨髓铁染色可见红细胞成串排列及大量环形铁粒幼细胞

根据患者的临床表现及相关实验室检查结果，经患儿父母知情同意，采集患儿外周静脉血行基因测序，结果显示，患儿ALAS2基因第4外显子的329-332位点TCAG缺失（c.329-332delTCAG），相应的氨基酸残基110处的缬氨酸变为丙氨酸，并在112个氨基酸终止编码（p.V110Afs*2）（图2A）。在人类基因突变数据库（The Human Gene Mutation Database，HGMD）中未见相关报道。Polyphen 2（http://genetics.bwh.harvard.edu/pph2/）软件预测中得分为0.00，该突变无害；SIFT（http://sift.jcvi.org）软件预测值为0.033，该突变对蛋白质功能影响不确定，然而本例患儿却有重度贫血，该变异不属于多态性位点，在人群中发生频率较低。采集其母亲外周血进行测序显示，患儿母亲为相应位点同样的杂合突变（图2B），患儿的上述变异遗传自母亲，可推断为致病性变异。结合遗传学、临床表现及病理学，综合分析诊断为XLSA。予维生素B_6_（100 mg/d）口服，治疗1个月后HGB水平明显升高（HGB 76 g/L、MCV 92.5 fl、MCH 22.7 pg、MCHC 245 g/L、RDW 24.8％），目前HGB已恢复正常。

**图2:**
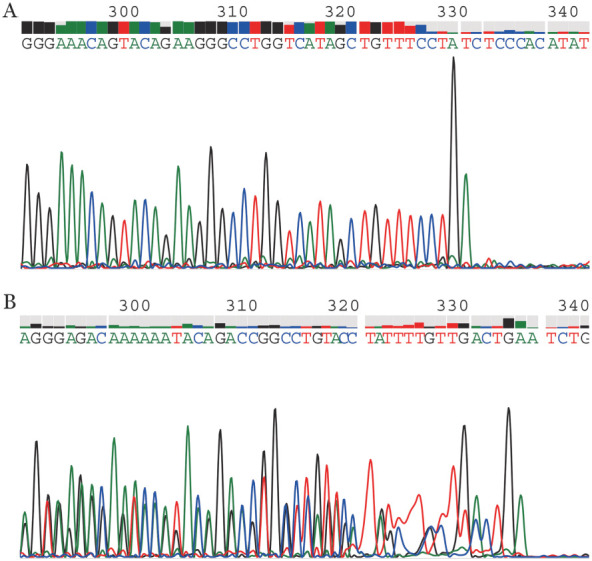
遗传性铁粒幼细胞贫血患者及其母亲ALAS2基因测序结果 A：患者ALAS2基因第4外显子c.329-332delTCAG（p.V110Afs*2），为半合子；B：患者母亲ALAS2基因第4外显子c.329-332delTCAG（p.V110Afs*2），为杂合子

## 讨论及文献复习

CSA是一组极为罕见的异质性疾病，由Cooley[4]于1945年首次报道，1992年Cotter等[5]在XLSA患者中首次发现ALAS2基因突变，并揭示了XLSA的致病基因。临床上，XLSA患者通常为半合子男性，发病年龄多<40岁，初诊断为不同程度的小细胞性贫血[3]，然而，也有中年XLSA病例的报道，多诊断为铁过载[6]。但也有少数男性不会发病[7]。大多数杂合子女性没有贫血的临床表现，因为正常的ALAS2蛋白可维持必需数量的红细胞产生，已报道的少量女性病例和家族性病例，可能是偏斜的X染色体失活和后天因素影响了ALAS2正常等位基因表达[8]–[9]。目前报道的104种ALAS2突变中，尚未见V110Afs*2。本研究为首次发现XLSA家系ALAS2基因V110Afs*2突变。

在血红素生物合成途径的第一步中，线粒体基质中的甘氨酸和琥珀酰辅酶A缩合成5-氨基乙酰丙酸（ALA），此反应由ALA合成酶催化，在反应过程中吡哆醛5′-磷酸（PLP，维生素B_6_）作为关键辅助因子，通过稳定琥珀酰部分的碳基和甘氨酸α-碳的相互位置，从而增强ALA合成酶的活性。随后，ALA被转运到细胞质中，参与血红素的合成[3]。Bailey等[10]报告了人类ALAS2晶体结构，ALAS突变后可引起ALAS蛋白构象发生改变，其C端延伸折叠到催化核心顶部形成loop结构，降低了对底物琥珀酰辅酶A的结合，从而影响ALA合成效率。此外，人类ALAS2与PLP采用一组高度保守的相互作用，主要涉及催化核心的中心子结构域。因此，我们推测PLP能够结合ALAS突变后形成的loop结构，抑制其进入ALAS酶活性中心顶部，由此增加ALAS酶与琥珀酰辅酶A的结合，从而增加ALA合成效率。人类ALA合成酶由ALAS2基因编码，当ALAS2基因发生功能丧失性突变时，ALAS2的酶活性降低，ALA不能有效生成，而转铁蛋白受体仍然稳定，因此摄入铁不能有效利用并积累在线粒体中。

XLSA患者的ALAS2基因突变通常是保守氨基酸的错义突变，通过改变ALAS2构象而导致功能丧失[3],[11]–[12]。在近一半的病例中，PLP治疗效果良好，从而恢复酶活性并改善小细胞低色素性贫血[10]。但仍有部分患者对吡哆醇治疗反应欠佳，ALAS2基因突变位点不同影响治疗反应：产生过早终止密码子的无义突变和使ALAS2蛋白失去稳定性的突变均会导致维生素B_6_治疗无效；启动子突变导致ALAS2基因转录调控受损会导致维生素B_6_疗效不佳[13]–[14]。对ALAS2基因突变的检测有助于准确诊断CSA及判断维生素B_6_疗效。维生素B_6_常用评价标准：①完全缓解：贫血治愈或接近治愈，HGB≥110 g/L；②部分缓解：贫血得到改善、血红蛋白水平升高>10 g/L或症状缓解；③无效：血红蛋白水平没有升高或升高≤10 g/L[15]。对于维生素B_6_难治性SA患者，可进行异基因造血干细胞移植，但疗效仍不确切[16]–[17]。

迄今为止，HGMD（http://www.hgmd.cf.ac.uk/ac/index.php）中登记的104种ALAS2突变中大多数与XLSA相关，且多位于第5～11外显子的高度保守区[6]，主要影响ALAS2的催化活性或者与PLP、甘氨酸或sCoA的结合[18]。在本病例中检测到的突变c.329-332delTCAG（p.V110Afs*2）位于第4外显子，该外显子突变的报道罕见。突变导致第110个氨基酸由甘氨酸变成丙氨酸，并在112个氨基酸终止编码。由于以前没有报道密码子110发生突变并且Val110不是已知的PLP结合位点，尚不清楚该突变如何影响PLP依赖性ALAS2活性，因此我们使用了SIFT和Polyphen-2来预测这种缺失突变的重要性，结果表明，SIFT评分为0.033，Polyphen-2评分为0。尽管预测分数较低，但V110A可能会产生功能改变的突变蛋白，从而引起XLSA发病，这种氨基酸取代的影响可能是间接的，但它可使蛋白质不稳定或影响酶催化作用[19]。患者ALAS2基因c.329-332delTCAG变异遗传来自其母亲，其母亲该位点为杂合子，符合X连锁遗传方式，为患者发病的致病性变异。患者母亲未发病，大多数女性携带者因ALAS2基因表达正常而不发病，但若出现X染色体失活，可出现X连锁疾病临床表现。由于患者采用维生素B_6_治疗有效，因此我们认为该突变可能影响ALAS2与PLP的相互作用。
